# Microglia specific deletion of miR-155 in Alzheimer’s disease mouse models reduces amyloid-β pathology but causes hyperexcitability and seizures

**DOI:** 10.1186/s12974-023-02745-6

**Published:** 2023-03-07

**Authors:** Macarena S. Aloi, Katherine E. Prater, Raymond E. A. Sánchez, Asad Beck, Jasmine L. Pathan, Stephanie Davidson, Angela Wilson, C. Dirk Keene, Horacio de la Iglesia, Suman Jayadev, Gwenn A. Garden

**Affiliations:** 1 Department of Laboratory Medicine and Pathology, School of Medicine, University of Washington, Seattle, WA 98195 USA; 2 Department of Neurology, University of Washington School of Medicine, Seattle, WA 98195 USA; 3 Department of Biology, University of Washington, Seattle, WA 98109 USA; 4 Department of Neurology, University of North Carolina at Chapel Hill, 170 Manning Drive, Chapel Hill, NC 27517 USA

**Keywords:** Alzheimer’s disease, Mouse models, miR-155, Microglia, Inducible knock-out, Epilepsy

## Abstract

Alzheimer’s Disease (AD) is characterized by the accumulation of extracellular amyloid-β (Aβ) as well as CNS and systemic inflammation. Microglia, the myeloid cells resident in the CNS, use microRNAs to rapidly respond to inflammatory signals. MicroRNAs (miRNAs) modulate inflammatory responses in microglia, and miRNA profiles are altered in Alzheimer’s disease (AD) patients. Expression of the pro-inflammatory miRNA, miR-155, is increased in the AD brain. However, the role of miR-155 in AD pathogenesis is not well-understood. We hypothesized that miR-155 participates in AD pathophysiology by regulating microglia internalization and degradation of Aβ. We used CX3CR1^CreER/+^ to drive-inducible, microglia-specific deletion of floxed miR-155 alleles in two AD mouse models. Microglia-specific inducible deletion of miR-155 in microglia increased anti-inflammatory gene expression while reducing insoluble Aβ_1-42_ and plaque area. Yet, microglia-specific miR-155 deletion led to early-onset hyperexcitability, recurring spontaneous seizures, and seizure-related mortality. The mechanism behind hyperexcitability involved microglia-mediated synaptic pruning as miR-155 deletion altered microglia internalization of synaptic material. These data identify miR-155 as a novel modulator of microglia Aβ internalization and synaptic pruning, influencing synaptic homeostasis in the setting of AD pathology.

**Supplementary Information:**

The online version contains supplementary material available at 10.1186/s12974-023-02745-6.

## Introduction

The pathological hallmarks of Alzheimer’s disease (AD) include the accumulation of extracellular Amyloid-β (Aβ) into plaques [1] and neuroinflammation [2]. An elevated risk of seizures also may be fundamentally connected to cognitive decline [3]. Variants in three genes, the Amyloid Precursor Protein (*APP*), Presenilin 1 (*PSEN1*) and Presenilin 2 (*PSEN2*), cause early onset familial AD (EOFAD; < 60 years of age), which overlap clinically and pathologically with late-onset sporadic presentations of AD (LOAD; > 60 years of age) [4, 5]. Patients with EOFAD have an 87-fold increased incidence of seizures relative to the age-matched population compared to a threefold increase in seizure incidence in LOAD individuals [6]. It’s currently unknown why EOFAD patients have higher risk of developing epilepsy, but the prevalence of comorbid seizures with AD suggests that AD pathology and network excitability are linked.

As observed in clinical AD, studies using transgenic mice expressing human APP have shown that high levels of Aβ peptide are sufficient to induce epileptiform activity and seizures even in the early stages of the disease process, leading to depletion of hippocampal stem cells and impairments in spatial discrimination [7, 8]. Spontaneous seizures and sharp-wave discharges have been observed in several transgenic models expressing human-APP. More recently, a relationship between Aβ production and epileptiform activity was established in the APP/PS1 transgenic mouse model as early as 4 months of age, prior to Aβ plaque deposits forming in the brain parenchyma [9, 10]. Therefore, network instability may precede complex plaque pathology and simpler Aβ species could be responsible for the elevated risk of seizures for AD patients.

Neuroinflammation is a significant contributor to AD progression but the precise role of key cellular modulators of inflammation is unclear. Microglia are the specialized resident myeloid cell population in the CNS that mediate innate immune responses [11, 12]. During acute inflammation, microglia maintain tissue homeostasis via debris containment, phagocytizing debris and misfolded or aggregated proteins, and initiating tissue repair signaling cascades that resolve pro-inflammatory activation [12]. In the diseased AD brain, however, several mechanisms may compromise the regulation of the microglia-mediated inflammatory response. The progressive deposits of Aβ fibrils into plaques create a positive feedback loop between inflammation and APP processing [13]. Accumulation of Aβ fibrils and neuronal debris that further activate receptors (TLRs, TREM2, CX3CR1) may establish a non-resolving, or chronic inflammatory state [14]. Therefore, understanding the mechanisms that modulate microglia-mediated neuroinflammation in the AD brain are key to understand circuitry changes and progressive cognitive decline.

A central mechanism mediating microglia inflammatory activation involves post-transcriptional modulation of inflammatory effectors. MicroRNAs (miRNAs) regulate the phasic responses of both developmental and physiological events [15]. As powerful epigenetic modulators of gene expression, miRNAs influence the timing and amplitude of the innate immune responses [15]. Several studies demonstrate that inflammatory miRNA profiles are altered in tissue, circulating monocytes and serum of AD patients. More specifically, certain miRNAs with inflammatory functions are dysregulated in the AD brain, including miR-155 [16, 17]. miR-155 can set the magnitude and timing of the pro-inflammatory response by targeting the 3’UTR of mRNAs that encode specific anti-inflammatory mediators [18, 19]. Mice deficient in miR-155 have a diminished ability to respond to inflammatory stimuli, like LPS [20, 21, 22], while mice overexpressing miR-155 develop chronic-inflammatory states and hematopoietic malignancies [23]. Expression of miR-155 is, therefore, required for normal immune function [24].

Previously, we demonstrated that miR-155 targets the anti-inflammatory transcription factor c-Maf for degradation in microglia [25]. Furthermore, miR-155 targets mRNAs coding for additional molecules involved in suppressing the inflammatory response including, SHIP1 [26] and SOCS1 [27], leading to increased pro-inflammatory gene expression. Recently, we identified a novel role for miR-155 in primary microglia, where conditional expression of miR-155 resulted in altered catabolism of fibrillar Aβ_1–42_ in vitro [28]. From this novel finding, we set out to understand the role of miR-155 in microglia, and how anti-inflammatory skewing of these cells impacted Aβ plaque pathology in AD mice. In this study, we observed that microglia-targeted inducible loss of miR-155 led to a reduction in total plaques at 6 months. We additionally identified a novel role for miR-155 in microglia, where inducible loss of miR-155 in microglia in vivo led to abnormal electrical activity, resulting in early-onset epileptogenesis, increased seizure frequency and duration, and increased mortality. Our findings suggest key overlap between Aβ pathology, network stability and vulnerability, and neuroinflammatory pathways modulated by miR-155.

## Methods

### Inducible microglia-specific knock-out of miR-155 in APP/PS1 and 5xFAD mice

All mice were maintained in a specific pathogen-free facility and group housed with ad libitum access to food and water on a 12/12 light dark cycle (lights on at 0600 h). Mice were maintained in a C57/Bl6 background and were handled based on an approved UW IACUC protocol and according to the Guide of the Use and Care of Laboratory Animals (3254-04). APPSwe/PS1dE9 (APP/PS1) or 5xFAD heterozygous mice were crossed with homozygous floxed miR-155 mice (miR-155^flx/flx^) to obtain homozygous miR-155^flx/flx^ alleles in the APP/PS1^±^ or 5xFAD^±^ background (miR-155^flx/flx^ APP/PS1^±^ or 5xFAD^±^). Mice with miR-155^flx/flx^ APP/PS1^±^ or 5xFAD^±^ were then crossed with miR-155^flx/flx^  CX3CR1^CreER/CreER^ mice to obtain trigenic APP/PS1^±^ miR-155^flx/flx^ CX3CR1^CreER/+^ or 5xFAD^± ^miR-155^flx/flx^ CX3CR1^CreER/+^ mice, and miR-155^flx/flx^ CX3CR1^CreER/+^ littermates. At 8 weeks of age, mice were treated with a one-time 20 mg dose of tamoxifen or corn oil (vehicle) by oral gavage. Tissue resident macrophages such as Kupffer cells, lung alveolar, splenic, and peritoneal macrophages, and microglia, that are established prior to birth maintain themselves through adulthood independent of replenishment by blood monocytes, therefore they retain the effects of the induced Cre [29]. Brains for the experiments delineated below were harvested at 6 months of age.

### Ex vivo FACS isolation of adult microglia and total RNA extraction.

Mice were anesthetized with Avertin (2.5%) until unresponsive to stimuli, then perfused with Hank’s Balanced Salt Solution (HBSS^−/−^) with 1 mM Hepes. The brain was extracted, hemisected, and the forebrain was placed in Accutase (Millipore, SRC005) to be mechanically processed and enzymatically dissociated for 30 min at 4 °C while shaking. Tissue was mechanically dissociated further with a serological pipette, then a micropipette, and strained through a 250 μm filter. The single cell suspension was resuspended in 100% Fetal Bovine Serum then in a 30% Percoll solution overlayed with FACS Media (10% FBS, 1 mM Hepes in HBSS^−/−^). The slurry was centrifuged at 800×*g* for 15 min with slow acceleration and break. The Percoll layer was then aspirated away from the pellet and cells were washed once in FACS Media. Cells were stained for CD45 expression with PE-Cy7-Rat anti-mouse CD45 antibody (BD Pharmigen; Clone 30-F11, catalog number: 552848) and with DAPI (Sigma). DAPI negative, CX3CR1-YFP^+^ and Pe-Cy7-CD45^int^ (microglia) cells were isolated and collected by FACS. Microglia were then pelleted at 2500 rpm at 4 °C for 5 min, then lysed in buffer from the DNA/RNA Mini-prep kit (Zymo;11-385). Lysed samples were frozen at − 80 °C until DNA/RNA was extracted.

### Detecting miR-155 deletion and changes in inflammatory effector genes targeted by miR-155

Total DNA and RNA were extracted from FACS isolated microglia using the DNA/RNA Mini-prep kit (Zymo; 11-385). DNA was used for end-point PCR to confirm the absence of miR-155 alleles. Total RNA was isolated and quantified using nano-drop, and samples that did not meet criteria (low concentration, 260:280 < 1.8) were excluded. Expression levels of mature miR-155-5p were detected using primer specific sequences for cDNA conversion using the TaqMan^®^ MicroRNA Assay for mmu-miR-155-5p (Assay ID 002571). cDNA from MISSION^®^ microRNA Mimics to hsa-miR-155 was used to generate cDNA for a standard curve to quantify total copy number of miR-155 per sample. To detect changes in gene expression of targets for miR-155, total RNA was used for cDNA conversion using random primers. cDNA was then used with the qPCR Roche primer/probe library to detect changes in *cMaf*  (Forward: 5′-CAACGGCTTCCGAGAAAAC-3′, Reverse: 5′-TCGCGTGTCACACTCACAT-3′), *Socs1* (Forward: 5′-TCTGTCTCCCCCATCAGC-3′, Reverse: 5′-GCGTGCTACCATCCTACTCG-3′), *Inpp5d * (*Ship1*; Forward: 5′-GGCTGAGGAGGACACTGTAGAA-3′, Reverse:, 5′-CGGCAGACATAGGAATGTT-3′), *Csf1r* (Forward: 5′-CCCTGATGTCAGAGCTGAAGA-3′, Reverse: 5′-TACAGGCTCCCAAGAGGTTG-3′) and *Tfeb* (Forward: 5′-CAACGATGAGATGCTCAGCTA-3′, Reverse: 5′-CTGTACACATCAAGTAGATTTCCAGAC-3′). Samples were normalized to a house keeping gene (*Gapdh;* Forward 5′-TGTGGAAGGGCTCATGACCA-3′, Reverse: 5′-CACCAGTGGATGCAGGGATG-3′) and Ct values were used in a delta–delta–Ct analysis to determine fold change in RNA levels (Quant Studio 6). Prism 7 (GraphPad) was used to graph changes in gene expression and run statistical analyses.

### Soluble and insoluble protein extraction and quantification from isolated structures of the mouse brain

The protocol as in [30] was followed for total protein extraction from frozen mouse cortex. Briefly, 150 μl of chilled RIPA buffer (1% NP-40, 0.5% sodium deoxycholate, 150 mM sodium chloride, 50 mM Tris hydrochloride, 0.5 mM magnesium sulfate; all from Sigma-Aldrich; St. Louis, MO) with Complete Mini protease inhibitor (Millipore Sigma-Aldrich; St. Louis, MO) was immediately added to the tube containing the sample once it was thawed and sitting on ice. Samples were then sonicated on ice (3 × pulses) and centrifuged for 30 min at 21,000×*g* and 4 °C. Supernatants containing RIPA-soluble proteins were pipetted off into new 2 mL tubes. The remaining pellet was washed with an additional 50 μl RIPA buffer with Complete Mini protease inhibitor and centrifuged a second time for 30 min at 21,000×*g* and 4 °C. The RIPA-buffer containing supernatant was then combined with the first RIPA-buffer containing supernatant. For Aβ_1–42_ extraction, 150 μl of chilled 5M guanidine–hydrochloride (Gu–HCl) buffer containing Complete Mini protease inhibitor was then added to the remaining pellet, vortexed, then sonicated (on ice for 3 pulses). Then, suspension was centrifuged at 13,000×*g* for 30 min at 4 °C to produce a Gu–HCl soluble supernatant containing the Aβ_1–42_ fraction and other RIPA insoluble proteins. All extracts were aliquoted and stored at − 80 °C. Total protein content was determined in all samples using a BCA kit (Pierce; Rockford, IL) with colorimetric detection (absorbance at 562 nm) in a plate reader. Total protein (75–150 ng as measured by the BCA assay) from each extract was added to wells of a Milliplex MAP kit HNABTMAG-68K and assayed for Aβ_1–40_ and Aβ_1–42_. Results from each sample were then normalized to total protein added to the well.

### In vivo continuous electrographic recordings

Mice were anesthetized with isoflurane and placed into a stereotaxic device where isoflurane anesthesia continued throughout surgery. A midline incision was made above the skull. Each mouse was implanted with ECoG electrodes consisting of dental screws (Pinnacle Technology, Lawrence, KS; No. 8209: 0.10-in.). Recording electrodes were screwed through cranial holes as follows: over the left frontal cortex (1.5 mm lateral and 2 mm anterior to bregma) and over the right parietal cortex (1.5 mm lateral and 2 mm posterior to bregma), a ground electrode was placed over the visual cortex (1.5 mm lateral and 4.0 mm posterior to bregma), and a reference electrode was placed over the cerebellum (1.5 mm lateral and 6.5 mm posterior to bregma). Electromyogram (EMG) signals were obtained by placing a pair of silver wires into the neck muscles. The screws were connected through silver wires to a common 6-pin connector compatible with the Pinnacle recording device. The screws and connector were fixed to the skull with dental cement. APP/PS1^±^ miR-155^flx/flx^ CX3CR1CreER^±^ and 5xFAD^±^ miR-155^flx/flx^ CX3CR1CreER^±^ mice were implanted at 7 weeks of age. Once the cap was fully dried and set (24 h) mice were fitted with a preamplifier and tether, and connected to the Pinnacle Technology recording system, where they were allowed 1 day to acclimate before recording started. The ECoG and EMG signals were sampled at 400 Hz with low-pass filters of 80 Hz and 100 Hz, respectively. Mice were connected to amplifiers where continuous recordings were made for 7 days to record a baseline reading. Mice were administered 20 mg of tamoxifen via oral gavage at 8 weeks. Continuous recordings persisted in single recording cages under a 12:12 LD cycle, with intermittent video, for 2–5 weeks until recording was stopped or when spontaneous death occurred.

### Interictal spike and seizure quantification

Interictal spikes (including spikes, polyspikes, and sharp waves) were manually labeled using a subset of data for both APP/PS1 and 5xFAD mouse models. A total of 95 time- and frequency-based features were extracted from signal data using 10-s epochs and normalized to zero mean and unit variance. Any epochs containing seizure data as well as the five epochs preceding and following the seizure event (respectively comprising the pre- and post-ictal periods) were removed.

We extracted features to fit linear discriminant analysis machine learning (ML) models using a least-squares solver and shrinkage estimated using the Ledoit–Wolf lemma. The output of the model was a binary decision (interictal spike present/not present). Two ML models were fit for APP/PS1 and 5xFAD mouse models separately, using only data from the corresponding mouse model, and used to predict presence of interictal spikes in signal data for the corresponding mouse model. Due to potentially unequal number of epochs for each animal and day, we converted binary model output to 1 (present) and 0 (not present) and took the mean value by day, thereby representing the percent of epochs in each day detected as having interictal spike activity. Specific days of interest were those representing one week prior to and one, two, and three (APP/PS1 only) weeks following oral gavage. Aside from the removal of epochs containing seizure activity, data from the entire day (i.e., 24 h) were used for prediction. Mixed-effects linear regression analysis was performed to determine the effect of time (i.e., hour) on interictal spike frequency to detect significant differences in global and group-level trends. Seizures were quantified manually through 24-h recordings at 7- 14- and 21-day post-tamoxifen treatment. Seizures (beginning of ictal phase to beginning of post-ictal depression period) were quantified per recording per animal monitored. Duration of the events (seconds) and total number of events were quantified for each animal before and after tamoxifen treatment.

### Identifying changes in plaque pathology in AD mice

Three sections of dorsal hippocampus, each 200 μm apart, were selected from each animal. Free floating sections were washed three times in 1X TBS then fixed for 20 min at room temperature in 4% PFA. After two 1X TBS washes, sections underwent a 20-min antigen retrieval in a sodium citrate buffer (pH = 6.0) at 65 °C and were cooled at room temperature for 20 min. After another two 1X TBS washes, sections were digested in 0.05% Proteinase K buffer for better plaque core visualization. After three 1X TBS washes, sections were incubated in blocking solution (1X TBS with 0.4% Triton X-100, 10% Donkey serum, 2% Bovine Serum Albumin, and 1% Glycine) at room temperature for 2 h. Sections were incubated in half-dilute blocking solution with primary antibodies (1:200; 6E10) for 18 h at 4 °C on shaker. Sections were washed three times in 1X TBST-T before secondary antibody (1:1000 Alexa 488 donkey anti mouse and 1:500 Alexa 594 donkey anti goat) incubation at room temperature for 3 h. DAPI (1:1000; Sigma) was added during last 30 min of secondary antibody incubation. After two final 1X TBS washes, sections were mounted with Vectashield containing DAPI and stored at 4 degrees in the dark until imaging. Observer was blinded to all animal conditions. Large-scale fluorescent images were taken with an inverted microscope. Channels across all images had identical exposure settings (100 ms DAPI channel, 800 ms FITC channel). Total plaques per section were counted manually, then data were graphed and analyzed using Prism.

### Quantifying internalized synaptic markers in ex vivo-isolated adult microglia

Mice were administered tamoxifen at 8 weeks of age as described previously. 2 week post-tamoxifen treatment, mice were deeply anesthetized with Avertin (2.5%) until unresponsive and then were perfused with cold Hank’s Balanced Salt Solution (HBSS^−/−^) and ex vivo isolation protocol was followed as described previously. Isolated cells suspended in 0.5 mL of FACS buffer were fixed with equal volumes of 4% PFA for 20 min at room temperature. Cells were washed in 0.5 mL of FACS buffer and pelleted at 2500 rcf for 5 min at 4 °C. Fixed cells were stored in 1 × PBS at 4 °C until ready to be stained. Cells in 1 × PBS were pelleted at 2500 rcf for 5 min at 4 °C and resuspended in 500 μL of fresh FACS buffer, then equally divided to be stained with anti-VGAT (Santa Cruz; sc-393373) and VGLUT1 (Abcam; ab77822) or negative control (DAPI only). Cells in Eppendorf tubes were placed overnight at 4 °C while gently shaking on an orbital shaker. The following day, cells were washed 2 × in 0.5 mL of FACS buffer by resuspending and re-pelleting at 2500 rcf for 5 min at 4 °C. Cells were then stained with secondary antibodies for 1 h at room temperature, lightly agitating each tube every 15 min, while protected from light. Cells were finally washed 2x (2500 rcf for 5 min at 4 °C) in 0.5 mL of FACS buffer to remove excess secondary antibody. Each sample was suspended in 350 μL of fresh FACS buffer for analysis using the LSR II/BD Diva software. Data were analyzed using FlowJo v10.0.

### Statistical analysis

FACS data were first analyzed to obtain mean fluorescence intensity values for the populations of interest. Statistics were run using R studio (version 1.4.1717). For all gene expression analyses, two-way ANOVAs were used with Tukey’s correction applied for multiple comparisons and main effects of treatment are reported, unless otherwise specified. A paired *t*-test was used to compare the amount of β-amyloid from insoluble and soluble fractions of adult brain, or plaque burden using histology. EEG spike data were quantified as described earlier. All results are displayed using mean and standard error of the mean.

## Results

### Inducible deletion of microglial miR-155 upregulates anti-inflammatory effector genes in isolated microglia from APP/PS1 mice

**Fig. 1:**
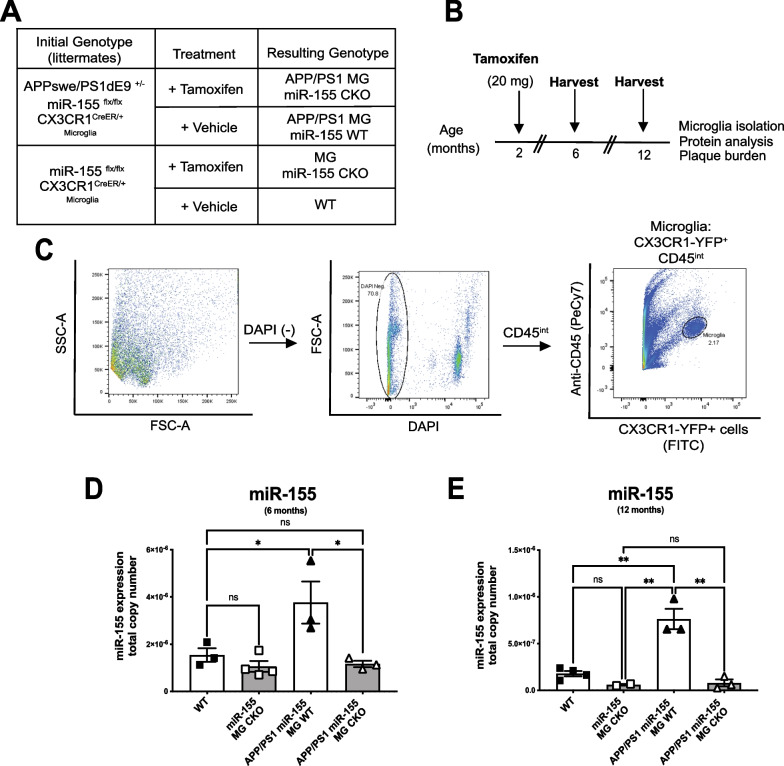
Expression of miR-155 is not detected at 12 months of age in ex vivo microglia from the APP/PS1 mouse model of AD after Cre-recombinase induction. **A** Experimental and control genotypes and groups. APPswe/PS1dE9 (APP/PS1) mice were crossed with miR-155flx/flx; CX3CR1^CreER^^/+^ mice to generate APP/PS1;miR-155flx/flx; CX3CR1^CreER/+^ (with tamoxifen: APP/PS1 MG miR-155 CKO mice; with corn oil: APP/PS1 MG miR-155 WT) or non-APP littermate controls that allow for conditional miR-155 deletion (Microglia miR-155 CKO). **B**) Experimental timeline of study. **C** Gating strategy for microglia isolation (CX3CR1-YFP^+^/ CD45^low^ cells) using ex vivo-FACS from the adult mouse CNS. miR-155 copy number in microglia from APP/PS1 MG miR-155 CKO mice and APP/PS1 MG miR-155 WT was quantified by qPCR at **D**) 6 months of age **E**) and 12 months of age (Stats: One-Way ANOVA with Tukey’s post hoc correction for multiple comparisons (*** = *p* < 0.0005)

To investigate the role of microglia miR-155 in the APP/PS1 mouse model of AD, we crossed APPSwe/PS1dE9^±^ (APP/PS1^±^) mice to generate APP/PS1^±^ miR-155^flx/flx^ CX3CR1^CreER/+^ mice and littermate controls (miR-155^flx/flx^ CX3CR1^CreER/+^) that do not express mutated human-APP (Fig. 1A). At 2 months of age, mice were given tamoxifen (20 mg) or corn oil (Fig. 1B) by oral gavage to achieve conditional, inducible knock-out (CKO) of miR-155 in microglia experimental mice (APP/PS1^±^ miR-155^flx/flx^ CX3CR1^CreER/+^) and littermate controls (miR-155^flx/flx^ CX3CR1^CreER/+^). Microglia (CX3CR1-YFP^+^/CD45^int^) cells were isolated using FACS (Fig. 1C) and total DNA and RNA was extracted from the isolated population. Successful deletion of miR-155 was confirmed using end-point PCR on genomic DNA from FACS isolated microglia. Deletion of miR-155 did not alter the ratio of microglia to total cells isolated by ex vivo-FACS (Additional file 1: Figure S1; two-way ANOVA with Tukey’s correction for multiple comparisons, main effect of genotype, *p* = 0.3923). Loss of miR-155 at the DNA level was still observed in microglia isolated from tamoxifen treated mice at 6 months of age, four months after tamoxifen treatment (not shown). We hypothesized that miR-155 expression would be increased in microglia isolated from old APP/PS1 mice. Using quantitative PCR (qPCR), we quantified miR-155 copy number in microglia isolated from 6-month-old and 12-month-old APP/PS1 mice compared to microglia from age-matched non-APP controls. We observed an increase in miR-155 copy number in APP/PS1^±^ miR-155^flx/flx^ CX3CR1^CreER/+^ that received corn oil (*p* = 0.0294) and wild-type mice (Fig. 1D; two-way ANOVA, with Tukey’s post hoc correction for multiple comparisons). In addition, we confirmed that treatment of in APP/PS1^±^ miR-155^flx/flx^ CX3CR1^CreER/+^ with tamoxifen resulted in a significant reduction of miR-155 copy number in microglia (*p* = 0.0162). (Fig. 1E; two-way ANOVA, with Tukey’s post hoc correction for multiple comparisons, main effect of genotype: *F*_(3,5)_ = 24.65, *p* = 0.0020. WT vs. APP/PS1 miR-155 MG WT, *p* = 0.0054). As expected, copy numbers of miR-155 were significantly reduced in APP/PS1^±^ miR-155^flx/flx^ CX3CR1^CreER/+^ tamoxifen treated animals compared to littermate vehicle controls (*p* = 0.0029). Animals that did not express the APP/PS1 transgene and received either tamoxifen or corn oil, did not show increased levels of miR-155 expression with age (*p* = 0.6120). These data show that inducible, Cre-mediated deletion of miR-155 in microglia results in a significant reduction in miR-155 expression at 6 months-of-age that is maintained late into adulthood.

**Fig. 2:**
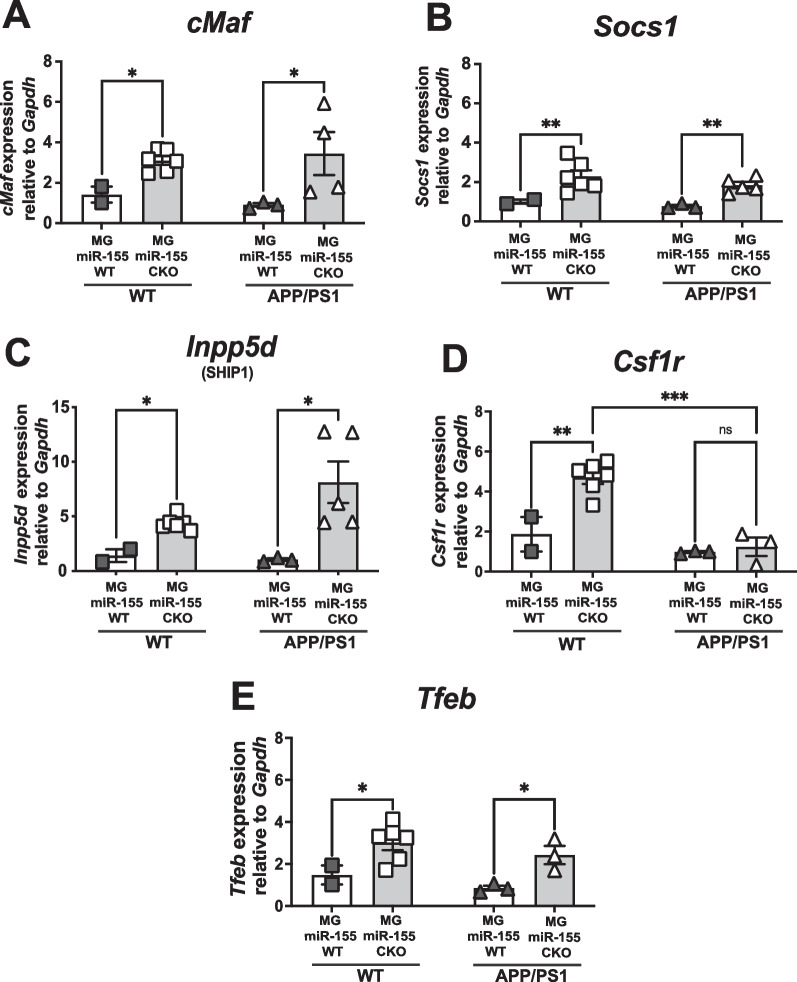
Anti-inflammatory gene expression is upregulated in microglia after microglia-specific knock-out of miR-155 in vivo. qPCR analysis of total RNA extracted form ex vivo-FACS sorted microglia (CX3CR1^YFP+^/CD45^int^ cells). In the APP/PS1 model **A**) *cMaf* **B**) *Csf1r* **C**) *Inpp5d* (SHIP1) **D**) *Socs1* and **E**) *Tfeb* are upregulated in microglia in vivo after miR-155 CKO. We observed that *Csf1r* was not significantly upregulated in microglia after miR-155 CKO in the APP/PS1 mouse model of AD (Stats: two-way ANOVA with multiple comparisons, Tukey’s post hoc correction (** = *p* < 0.005, *** = *p* < 0.0005)

Since a single miRNA can modulate expression of many effector genes, we focused on identifying changes in expression of genes that are confirmed or interesting putative targets for miR-155. Amongst the many targets of miR-155 targets of interest relevant to AD pathogenesis are anti-inflammatory effectors, genes that are required for microglia survival, proliferation, and lysosomal biogenesis are of interest, as these are pivotal biological pathways dysregulated in AD. We quantified changes in gene expression using total RNA extracted from FACS-isolated microglia of APP/PS1 and littermate control mice at 6 months of age, 4 months after tamoxifen treatment and when amyloid pathology is observable in the model. We focused on changes in expression of miR-155 target genes: c*Maf* (cMAF), *Socs1* (SOCS1), *Inpp5d* (SHIP1), and *Csf1r* (CSF1R). At 6 months of age, *cMaf* (*p* = 0.0059), *Inpp5d* (*p* = 0.0028), and *Socs1* (*p* = 0.0012) were upregulated in miR-155 deleted microglia isolated from both APP/PS1 mice and non-APP/PS1 littermates; Fig. 2A–D; two-way ANOVA with Tukey’s post hoc correction for multiple comparisons, main effect of treatment). Interestingly, we observed an interaction between treatment and genotype in *Csf1r* expression. While *Csf1r* was upregulated in non-AD littermates after Cre-mediated induced knock-out of miR-155 relative to vehicle controls (*p* = 0.0052), *Csf1r* expression remained unchanged in APP/PS1 microglia with miR-155 loss (*p* = 0.9755) and expression of *Csf1r* was significantly different from non-AD littermates (*p* = 0.0004). With the exception of *Csf1r*, we conclude that several miR-155 target genes with known anti-inflammatory function are upregulated in microglia at 6 months of age after microglia specific deletion of miR-155 in APP/PS1 mice.

*Tfeb* is a putative target for miR-155 that we identified via a series of in silico alignments using the miRanda web-based miRNA Target Prediction tool. This transcription factor regulates expression of lysosome biogenesis genes [31]. The endosome/lysosome system is involved in microglia-mediated degradation of internalized Aβ [32, 33] and multiple genetic risk alleles associated with AD are thought to be active in this pathway [34]. *Tfeb* was significantly upregulated in APP/PS1 and littermate control microglia after miR-155 deletion (Fig. 2E two-way ANOVA with Tukey’s post hoc correction for multiple comparisons, main effect of treatment, *p* = 0.0055). This finding suggests that miR-155 suppresses *Tfeb* expression in mouse microglia, a pathway by which chronic inflammation may prevent microglia mediated degradation of Aβ.

Previous studies have suggested that a subset of mouse microglia isolated from disease model brains demonstrate a specific pattern of gene expression referred to as disease associated microglia (DAM) and that miR-155 could contribute to gene expression changes associated with the DAM phenotype [35]. We utilized qPCR to detect changes in gene expression profiles of DAM-associated genes (Additional file 2: Figure S2). We did not observe significant differences in *Iba-1* (2A: Ordinary one-way ANOVA with Sidak’s correction for multiple comparisons, *p* = 0.4366), *Cst3* (2C: Ordinary one-way ANOVA with Sidak’s correction for multiple comparisons, *p* = 0.7507) or *Hexb* (2D: Ordinary one-way ANOVA with Sidak’s correction for multiple comparisons, *p* = 0.6188) between our three different groups. This is not unexpected, since the population of DAM microglia is a small fraction relative to the total microglia population in a variety of disease models. *Tmem119* expression was reduced in APP/PS1 microglia relative to control and miR155 CKO reversed this change (2B: Ordinary one-way ANOVA with Sidak’s correction for multiple comparisons, *p* = 0.0266, Control v. APP/PS1: *p* = 0.0251, APP/PS1 MG miR-155 CKO vs. APP/PS1:* p* = 0.1921). These finding suggest that impact of miR-155 CKO in microglia may not be specific to the regulation of the DAM pattern of gene expression change.

### Inducible deletion of miR-155 from microglia leads to decreased insoluble Aβ_1-42_ and reduced plaque pathology in APP/PS1 mice

**Fig. 3:**
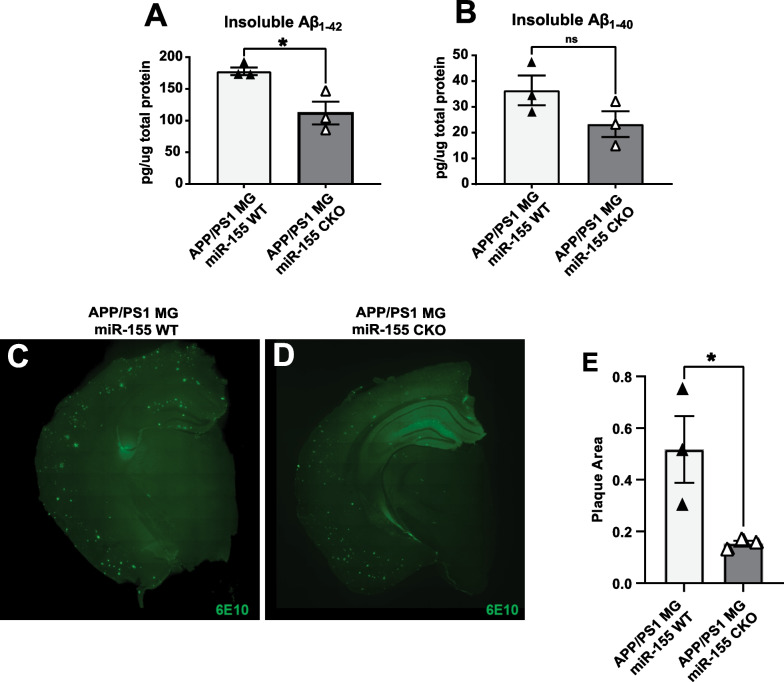
miR-155 deletion from microglia in adult APP/PS1 mice leads to a reduction in insoluble Aβ_1–42_ and total plaque area. **A** Analysis of the insoluble protein fractions by Luminex detected decreased levels of Aβ_1–42_ in APP/PS1 microglia miR-155 conditional knock-out mice (APP/PS1 MG miR-155 CKO) compared to non-deleted controls (APP/PS1) (two-tailed unpaired *t*-test, * = *p* < 0.05), **B** while there was no detectable change in Aβ_1–40_ levels. **C** and **D** Representative images of immunostaining for Aβ-plaques (6E10) in APP/PS1 and APP/PS1 miR-155 MG CKO brains. **E** Aβ-plaque area quantification based on 6E10 immunostaining in CA1 and CA3 (two-tailed unpaired *t*-test, * = *p* < 0.05)

We previously reported that conditional knock-out of miR-155 in cultured neonatal microglia led to increased catabolism of fibrillar forms of Aβ_1–42_ [28]*.* In the APP/PS1 mouse, we observed that conditional knock-out of miR-155 in microglia resulted in upregulation of several anti-inflammatory miR-155 target mRNAs, including a novel putative target, *Tfeb*. Therefore, we asked if microglia-specific deletion of miR-155 altered total levels of Aβ_1–42_ in vivo*.* Using Luminex-based assays, we quantified total levels of Aβ_1–42_ and Aβ_1–40_ from insoluble protein fractions isolated from the cortex of 6-month-old APP/PS1 mice post-microglia-specific knock-out of miR-155 at 2 months of age. We found that total levels of insoluble Aβ_1–42_ were reduced approximately 30% in cortex of APP/PS1 mice after miR-155 knock-out in microglia compared to control APP/PS1 mice of the same cohort (Fig. 3A; two-tailed unpaired *t*-test, *p* = 0.02645). Aβ_1–40_ from insoluble protein fractions also trended downward in APP/PS1 mice after miR-155 knock-out microglia but did not reach statistical significance (Fig. 3B; two-tailed unpaired *t*-test, *p* = 0.1607). As expected, there was no significant difference in the levels of soluble Aβ_1–42_ and Aβ_1–40_ in cortical lysates (Additional file 3: Figure S3). We did not observe significant differences between APP/PS1 and APP/PS1 mice after miR-155 knock-out in microglia in hippocampal lysates (data not shown). Levels of pTau are not high in the APP/PS1 mouse model [36]; however, we did quantify pTau levels using Luminex bead-based assays. There was no significant change in cortical or hippocampus pTau after microglia-specific miR-155 deletion (Additional file 4: Figure S4A; two-tailed unpaired *t*-test, *p* = 0.7858, B; two-tailed unpaired *t*-test, *p* = 0.1927) in APP/PS1 mice at 6 months of age. Taken together, conditional knock-out of miR-155 in microglia results in reduced insoluble Aβ_1–42_ in APP/PS1 mice.

We next asked if conditional knock-out of miR-155 in microglia led to alterations in established plaque pathology. We hypothesized that conditional miR-155 knock-out in microglia would lead to decreased plaque burden in older animals with established plaque pathology. We induced miR-155 deletion in microglia at 8-months of age in APP/PS1 mice, an age where amyloid plaque pathology is easily observed using antibodies (anti-6E10) directed against Aβ fibrils. Then, we quantified changes in plaque pathology two weeks after miR-155 knock-out. We observed a global decrease of plaque area in CA1 and CA3 in hippocampus of APP/PS1 mice 2 weeks after inducible miR-155 knock-out in microglia compared to age matched AD mice (Fig. 3C, D are representative images, quantification shown in 3 E; unpaired two-tailed *t*-test, *p* = 0.04797). We did not observe a significant difference in total number of plaques (Welch’s two-tailed *t*-test, *p* = 0.1246, data not shown). Therefore, inducible microglia-specific loss of miR-155 in APP/PS1 results in a decrease of plaque area in hippocampus of 8-month-old APP/PS1 mice, suggesting a potential role of pathways regulated by miR-155 in plaque clearance and Aβ catabolism in vivo.

### Microglia-specific and inducible deletion of miR-155 results in early mortality and decreased overall survival of APP/PS1 mice

**Fig. 4:**
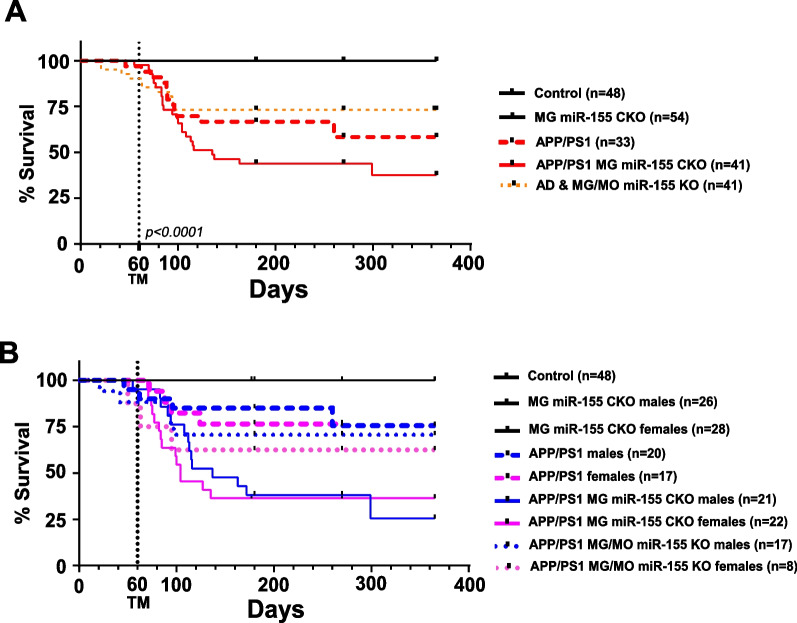
Microglia-specific inducible knock-out of miR-155 leads to increased mortality in the APP/PS1 mouse model of AD. **A** Survival analysis of experimental and control lines and littermate controls revealed significant mortality in APP/PS1 mice after microglia-specific deletion of miR-155 (Stats: Log-Rank test with pairwise comparisons and Bonferroni’s post hoc correction). **B** There was no sex-specific difference in survival observed within genotypes (Stats: Log-Rank test based on sex, *p* = 0.5)

We hypothesized that microglia-specific knock-out of miR-155 in the APP/PS1 mouse model of AD would lead to upregulation of anti-inflammatory profiles of microglia, reduced amyloid pathology, and therefore increased survival of the mouse model. However, we observed that inducible microglia specific deletion of miR-155 led to a significant increase in mortality of APP/PS1 mice (53%) as early as 3 months of age (Fig. 4A. APP/PS1 v. APP/PS1 MG miR-155 CKO, *p* = 0.04629) Log-Rank test with Bonferroni post hoc correction for pairwise comparisons based on genotype (see Additional file 6: Table S1). Loss of miR-155 in microglia in the non-APP/PS1 littermate controls did not have an impact on survival. Necropsies demonstrated no gross anatomic abnormalities in peripheral organs that could suggest an alternate non-neurological mechanism contributing to the spontaneous death phenotype. In addition, we observed that constitutive, developmental loss of miR-155 in all CX3CR1-expressing cells (microglia in the CNS and macrophages in the periphery) did not impact the long-term survival of APP/PS1 mice (APP/PS1 v. APP/PS1 MG/MO miR-155 KO,* p* = 0.59652), as did the inducible loss of miR-155 in microglia (Fig. 4A). There was no influence of sex on survival of APP/PS1 line with inducible knock-out of miR-155 in microglia or with developmental loss of miR-155 in all CX3CR1-expressing cells (Fig. 4B; Log-Rank test based on sex, *p* = 0.5). Taken together, we conclude that inducible loss of miR-155 expression in microglia is sufficient to increase spontaneous death in the APP/PS1 mouse model of AD by a novel mechanism.

### Inducible deletion of miR-155 in microglia leads to early onset hyperexcitability and seizures that result in spontaneous death

**Fig. 5:**
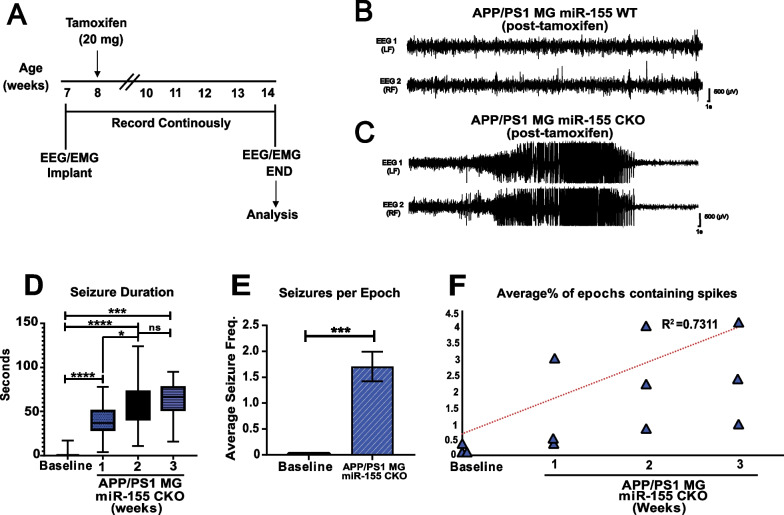
Microglia-specific inducible knock-out of miR-155 leads to increased seizure burden in the APP/PS1 mouse model of AD. **A** Experimental design summary. Mice were implanted with ECoG and EMG electrodes at 7 weeks of age and, after a baseline recording, miR-155 knock-out was induced at 8 weeks. Continuous ECoG recordings were done for 2–5 weeks or until spontaneous death. Representative trace of **B** baseline and **C** seizure that resulted in a spontaneous death event. **D** Spontaneous seizures were identified and manually quantified starting 1-, 2- and 3-week post-miR-155 deletion in microglia. (Stats: Mixed-effects ANOVA with Tukey’s post hoc correction: ****p < 0.0001, ***p < 0.001, *p < 0.01). **E** Seizure frequency was increased post miR-155 deletion in microglia in the APP/PS1 background (Stats: Wilcoxon rank sum test with continuity correction, ***p < 0.00001). **F** % epochs (hours) containing inter-ictal spikes, high-amplitude, synchronous spiking observable via cortical ECoG were increased after miR-155 deletion in microglia (Stats: Mixed Linear Model, *p* < 0.0001, *R*^2^ = 0.7311)

Spontaneous death in APP/PS1 mice is often associated with seizures, a well-known phenomenon in human-APP transgenic mice. Hyperexcitability and seizures were observed as early as 4.5 months of age in APP/PS1 mice [37]. We observed a 53% mortality rate after inducible deletion of miR-155 specifically in microglia within the first 6 months of age in our APP/PS1^±^ miR-155^flx/flx^ CX3CR1^CreER/+^ mice. Therefore, we decided to identify changes in hyperexcitability prior to and immediately post deletion of miR-155 in microglia in vivo. We hypothesized that the inducible loss of miR-155 in microglia resulted in a further compromised circuitry leading to early onset epileptogenesis and spontaneous death. To characterize the seizure profile resulting from inducible deletion of miR-155 in microglia, we implanted APP/PS1^±^ miR-155^flx/flx^ CX3CR1^CreER/+^ mice at 7 weeks of age with recording electrodes to assess cortical activity over frontal and parietal cortices (Fig. 5A). We recorded a 7-day baseline EEG/EMG with intermittent video recording. Prior to miR-155 deletion in microglia, we observed basal EEG during wake and sleep periods (Fig. 5B; representative trace) that appeared unchanged from traces obtained from wild-type mice. After tamoxifen treatment via oral gavage at 8 weeks of age, we observed a significant increase in spontaneous, electrographic, and behavioral seizures as early as 7 day post-miR-155 knock-out in microglia. We continuously recorded electrographic activity for 3–5 weeks post-miR-155 deletion in microglia or until a spontaneous death event was captured on ECoG (Fig. 5C; representative trace). Spontaneous recurrent seizures lead to sudden death in two-thirds of the cohort recorded. Inducible loss of miR-155 in microglia resulted in increased seizure duration for at least 3 week post-miR-155 knock-out in microglia (Fig. 5D; Ordinary one-way ANOVA with Tukey’s post hoc correction for multiple comparisons: *F*_(3, 131)_ = 64.45, *p* < 0.0001). Seizure frequency per recording period of 24 h was also significantly increased in APP/PS1 mice after miR-155 knock-out in microglia (Fig. 5E; seizure frequencies are not normally distributed and show unequal variance, Wilcoxon rank sum test with continuity correction, *p* = 1.493e-07). As expected, interictal spikes per hour were significantly increased during both sleep and wake periods, supporting increased hyperexcitability as early as 1 week after miR-155 loss in microglia, and continuing throughout the study (Fig. 5F, Mixed Linear Model, *p* < 0.0001, *R*^2^ = 0.7311). We did not observe inter-ictal discharges or seizures in wild-type mice (not shown). We conclude that inducible loss of miR-155 in microglia leads to increased seizure severity (frequency and duration) as early as 7 day post-miR-155 deletion that progresses and is maintained throughout the recording period. Therefore, increased hyperexcitability, seizure burden, and sudden death prior to the onset of histologically detectable plaque pathology in APP/PS1 mice are pathophysiological processes sensitive to mechanisms that converge on miR-155 regulation of microglia function.

### Microglia-specific deletion of miR-155 causes hyperexcitability in the 5XFAD mouse model of AD

**Fig. 6:**
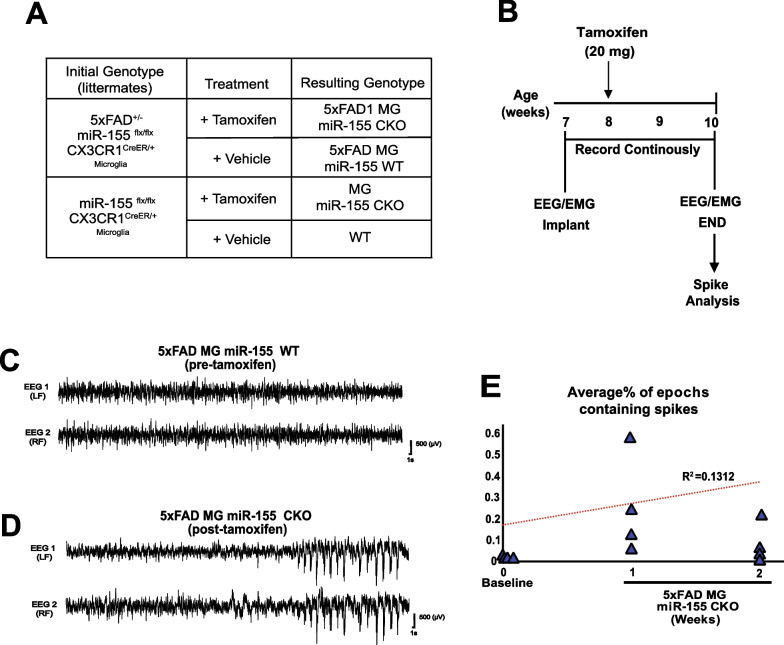
Microglia-specific inducible knock-out of miR-155 in leads to aberrant cortical excitability in the 5xFAD mouse model of AD. **A** Experimental and control genotypes and groups. **B** Experimental design summary. Mice were implanted with ECoG and EMG electrodes at 7 weeks of age, and after a baseline recording, miR-155 knock-out was induced at 8 weeks. Continuous recordings were made for 2 weeks. Representative trace of **C** baseline and **D** high-amplitude, synchronous spiking in the EEG. **E** %epochs (hours) containing inter-ictal spikes, high-amplitude, synchronous spiking in the EEG were increased after miR-155 deletion in microglia in 5xFAD mice (Mixed Linear Model, *p* = 0.027, *R*^2^ = 0.1312)

We previously observed that microglia specific deletion of miR-155 in APP/PS1 mice resulted in increased seizure frequency, hyperexcitability, and spontaneous death. We next asked if miR-155 deletion in microglia led to changes hyperexcitability in a second amyloidosis model of AD, in the absence of plaque pathology. We crossed 5xFAD^±^ mice to miR-155^flx/flx^ and CX3CR1^CreER/+^ mice obtain 5xFAD miR-155^flx/flx^ CX3CR^Cre^^ER/+^ allowing us to conditionally delete miR-155 from microglia in the 5xFAD amyloidosis model (Fig. 6A). We implanted 7-week-old mice with screws/silver wire electrodes (Fig. 6B) and recorded a baseline ECoG/EMG (with intermittent video) for one week. We then induced miR-155 deletion at 8 weeks of age with tamoxifen treatment. We recorded from mice continuously for 2 weeks post deletion of miR-155 in microglia (Fig. 6C; representative traces). Interictal spikes in 5xFAD were increased as early as 1 week after microglia miR-155 deletion (Fig. 6D; representative trace), as seen in the APP/PS1 line. This increase in interictal spikes was significantly different from baseline throughout the 2-week recording period (Fig. 6E; Mixed Linear Model, *p* = 0.027, *R*^2^ = 0.1312). Hyperexcitability in the 5xFAD mice was observed prior to development of mature plaque pathology (Additional file 5: Figure S5). These findings suggest that the loss of miR-155 in microglia is sufficient to increase hyperexcitability in the context of two distinct AD models.

### Inducible miR-155 deletion in AD mice results in more excitatory synaptic marker internalization by microglia

**Fig. 7:**
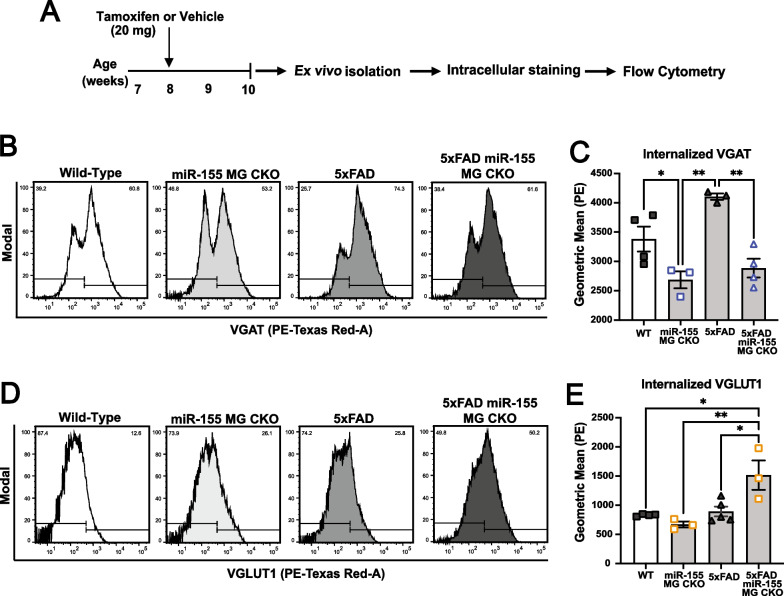
Inducible knock-out of miR-155 in microglia results in increased internalization of excitatory synaptic terminals marked by VGLUT1 in the 5xFAD brain. **A** Microglia were isolated using ex vivo, then fixed and stained for the pre-synaptic vesicular GABA transporter (VGAT; representative histograms **B** in and quantification in **C**), and the pre-synaptic vesicular glutamate transporter 1 (VGLUT1; representative histograms **D** in and quantification in **E**). Total levels of internalized VGAT and VGLUT1 were detected with flow cytometry (LSRII). Data were analyzed using FlowJo and R studio, graphs were generated using Prism 9. (Stats: two-way Ordinary ANOVA with multiple comparisons with Tukey’s post hoc corrections, ****p < 0.0001, ***p < 0.001, **p < 0.01)

Circuitry disruption in the balance between excitatory and inhibitory inputs can create a hyperexcitable state, increased susceptibility for seizure generation, and epileptogenesis. To begin to understand how microglia contribute to circuit disruption, we tested the hypothesis that microglia may aberrantly prune excitatory or inhibitory synaptic terminals in the context of Aβ. We isolated adult microglia by Percoll density gradient 2 weeks after deletion of miR-155 in vivo when we observed changes in cortical excitability, and stained for internalized inhibitory or excitatory pre-synaptic markers (Fig. 7A). We detected microglia immunolabeled for the internalized inhibitory synaptic terminals VGAT (Fig. 7B; representative histograms of VGAT^+^ signal), and internalized excitatory synapses marked by VGLUT1 (Fig. 7D; representative histograms of VGLUT1^+^ signal) using flow cytometry. Microglia from tamoxifen treated 5xFAD miR-155f^lx/flx^ CX3CR1^Cre^^ER/+^ mice that lacked miR-155 internalized significantly less VGAT-labeled synapses compared to corn oil (control) treated animals (Fig. 7B, C. Two-way ANOVA with multiple comparisons with Tukey’s post hoc correction: *F*_(3,7)_ = 14.96, *p* = 0.0020. For 5xFAD and 5xFAD MG miR-155 CKO, *p* = 0.0073). Interestingly, 5xFAD microglia demonstrate increased VGAT synapse internalization compared to wild type microglia, though this difference is not statistically significant post hoc (*p* = 0.1151 with Tukey’s post hoc).

We observed a significant increase in hyperexcitability and seizure-related mortality after deletion of miR-155 in microglia in the APP/PS1 line. Since we observed reduced VGAT internalization after miR-155 deletion, we asked if microglia were selectively removing excitatory synaptic terminals*.* We observed a significant increase in VGLUT1 internalization by microglia from miR-155 deleted 5xFAD brain compared to all other groups (Fig. 7D and E) (Two-way ANOVA multiple comparisons with Tukey’s post hoc correction: *F*_(3,7)_ = 9.100, *p* = 0.0082. 5xFAD: *p* = 0.0205; miR-155 MG CKO: *p* = 0.0083; WT: *p* = 0.0215). There was no significant increase in microglia VGLUT1 internalization after miR-155 deletion in non-AD controls (*p* = 0.7733) or in 5xFAD microglia (*p* > 0.999) relative to wild type. Thus, in the context of Aβ, specific miR-155 deletion in microglia leads to increased internalization of excitatory synapses and decreased internalization of inhibitory synapses. Imbalance between excitatory and inhibitory synaptic activity can lead to hyperexcitability and seizures. For example, loss of excitatory currents due to mutations in NaV_1.1_ (*Scn1a*) in Dravet Syndrome results in epilepsy. While loss of excitatory activity resulted in seizures seemed counterintuitive, genetic mouse models of Dravet Syndrome demonstrate that NaV1.1 loss predominantly impacts inhibitory circuitry leading to hyperexcitability [38]. Similarly, our findings suggest that altering microglia internalization of synapses in the context of Aβ may have unpredictable impact on the overall balance between synaptic inputs. Our findings support the hypothesis that miR-155 influences how microglia actively modulate excitatory and inhibitory synaptic balance, contributing to hyperexcitability and seizures in AD.

## Discussion

A growing number of studies have highlighted the significance of inflammatory miRNAs as epigenetic modulators of microglia cellular functions in senescence, cellular aging, and in neurodegenerative diseases, like AD [39, 40]. Thus, current efforts in the field are focused on understanding how miRNAs modify inflammation, cellular behaviors, and intra-cellular crosstalk in AD pathophysiology. Experimental mouse models of AD have been employed to elucidate changes in miRNA expression in vivo [41, 42, 43, 44]. Dysregulation of miR-155 has been repeatedly linked to neurodegeneration and AD [35, 45]. In mouse the 3xTg AD model, early upregulation of miR-155 is observed in situ, prior to development of Aβ plaques [45]. In the APP/PS1 mouse, we observed that isolated mature microglia show increased expression of miR-155 compared to age matched controls, suggesting dysregulation of miR-155 expression in this model in late adulthood. In addition, in this work, we demonstrate a reduction of insoluble Aβ_1–42_ and plaque pathology after miR-155 deletion in microglia. Our work supports previous findings of a role for miR-155 in modulating catabolism of Aβ both in vitro [28] and now, in vivo. Our study clearly shows that inflammatory miR-155 modulation of microglia functions is pivotal in AD pathophysiology, and that a more nuanced role for miRNA regulation in microglia impacts synapse engulfment and excitability in early pathogenesis.

The pro-inflammatory functions of miR-155 have been studied in various models of disease, including AD and ALS mice. In ALS (B6/SJL-SOD1^G93A^ transgenic) mice it has been shown that germ-line knock-out of miR-155 altered microglia polarization in vivo [46]*.* Increased levels of miR-155 promotes pro-inflammatory cytokine secretion by targeting the Suppressor of Cytokine Signaling 1 (SOCS1) message for degradation and downregulating anti-inflammatory cytokines that act via the JAK/STAT pathway [27, 47] and NF-kB signaling [48]. As one of the five miRNAs located on chromosome 21 flanking the APP gene, the pathological implications of additional copies of miR-155 extend beyond inflammatory regulation and have gained interest and attention in Trisomy-21 and AD pathogenesis [49, 50]. In our work, we used a microglia-specific inducible Cre-Lox system (miR-155 ^flx/flx^ CX3CR1^Cre^^ER/+^) and observed that inducible loss of miR-155 expression in mature microglia in the APP/PS1 model resulted in the expected upregulation in anti-inflammatory gene expression of the majority of its established mRNA targets in the APP/PS1 model and in non-APP/PS1 littermate controls. Interestingly, we did not observe a significant difference in expression of *Csf1r* with loss of miR-155 at the time of induction in the APP/PS1 model relative to non-APP/PS1 and vehicle controls. The colony stimulating factor 1 receptor (*Csf1r*) is required for the development, maintenance, and proliferation of microglia, and dysregulation of CSF1R/IL-34 signaling contributes to changes in density and distribution of microglia populations in the mouse brain [51]. While turnover of microglia under homeostatic conditions in the brain is low, in APP/PS1 mice proliferation of microglia was threefold [52]. Our data suggest that *Csf1r* expression is suppressed in adult microglia in the APP/PS1 brain and is unaltered with miR-155 loss. However, a limitation of our study is that we did not quantify differences in microglia population dynamics, either in microglia number or turnover, resulting from miR-155 loss in the APP/PS1 brain. Since senescence and inflammatory expression profiles are increase with age and are brain region- and sex-specific [53], the effect of miR-155 modulation in the aged APP/PS1 brain requires further study. Future studies should focus not only on the changes in inflammatory gene expression in microglia in vivo as a result of miR-155 loss, but also on the impact of miR-155 loss in microglia population dynamics and other compensatory mechanisms potentially mediated by astrocytes.

We identified novel pathways regulated by miR-155 that lead to increased Aβ catabolism in vivo, excitatory synaptic removal, as well as early onset of hyperexcitability. With this in mind, we used in silico approaches to identify novel targets for miR-155. With a series of alignments to predict miRNA binding sites, we identified that *Tfeb*, transcription factor and modulator of lysosome biogenesis [31], may be targeted by miR-155. We observed that expression of *Tfeb* was significantly increased with inducible loss of miR-155 in adult microglia isolated from the APP/PS1 and non-APP/PS1 mouse brain. Additional studies are needed to validate *Tfeb* mRNA as a direct target of miR-155, as our findings suggest this may be a novel pathway regulating Aβ catabolism by microglia. Future work should also focus on elucidating the mechanisms of action through which miR-155 influences microglia mediated synaptic pruning. This is important since deletion of miR-155 substantially ameliorated Aβ pathology but concurrently caused circuitry changes leading to hyperexcitability and seizures.

In AD patients and experimental models, microglia closely associate with amyloid plaques, exhibiting an ameboid morphology suggestive of inflammatory activation. Studies using radiolabeled or fluorescent Aβ [54] and direct injection of fibrillar Aβ into rat brains [55] demonstrated the capability of microglia to internalize Aβ in the rodent brain. More recently, the transcriptional signature of Aβ-plaque associated microglia has revealed significant differences in inflammatory regulation and transcriptional signatures associated with accelerated ageing [56], and therefore, we focused the role of inflammatory miRNAs, like miR-155, in AD pathogenesis. We observed that inducible loss of miR-155 in primary microglia in vitro resulted in increased catabolism of Aβ_1–42_ while overexpression of miR-155 led significantly reduced catabolism [28]. In the APP/PS1 mouse we observed a significant reduction in cortical insoluble Aβ_1–42_ and total plaque load in mice after miR-155 loss in APP/PS1 mice relative to vehicle controls. Previous studies have shown that cell bodies of microglia in AD model mice were larger and displayed ultrastructural signs of cellular stress, especially those nearby plaques and that these cells were overall less phagocytic [57]. In addition, in areas with limited plaque pathology, microglial processes encircled synaptic elements more often compared with plaque-associated processes [57]. Therefore, future studies focused on understanding the diversity of microglial responses to fibrillar and plaque Aβ_1-42_ pathology and synaptic loss upon loss of miR-155 are needed.

A role of miR-155 in epileptogenesis has been reported. Increased levels of miR-155 are observed in tissue isolated from Temporal Lobe Epilepsy (TLE) patients [58]. In a rat model of TLE, antagomir administration to inhibit miR-155 epigenetic regulation on target mRNAs led to a reduction in the pathophysiological features of TLE [59]. A different model of kainic acid induced seizures showed that silencing of miR-155 attenuated seizures via a microglia-mediated mechanism [60]. However, the impacts of miR-155 regulation specifically in microglia were not investigated in these models. A third study aimed at identifying a mechanism impacted by miR-155 expression, where miR-155 was identified to regulate on mTOR levels in TLE patient tissue and mouse models. Expression of miR-155 modulated expression of BDNF and TrkB [61] while intranasal delivery of an miR-155-5p antagomir alleviated acute seizures by countering hippocampal inflammation in pentetrazol-induced model [62]. Our study is the first to identify a role in hyperexcitability and epileptogenesis for miR-155, where loss of miR-155 specifically in microglia resulted in early onset hyperexcitability, increased seizure frequency and burden, and increased mortality in AD mouse models.

It is estimated that 10–22% of patients with AD develop spontaneous, unprovoked electrographic seizures [3, 63]. Familial and early onset AD patients have a higher risk of developing seizures [64]. Patients with AD and seizure disorders show greater cognitive impairment, faster progression of symptoms, and more severe neuronal loss at autopsy than those without seizures [6, 64, 65]. The identity of the cellular players and molecular mechanisms contributing to hyperexcitability in dementias remain largely obscure. Recent reports suggest both cell autonomous and non-cell autonomous mechanisms are contributors to epilepsy in AD [7, 10]. Specifically, there is a strong correlation between plaque number and the increase of seizure severity in mice overexpressing human APP (APP/PS1) [66]. Moreover, APP/PS1 mice present with a lower seizure threshold when challenged with chemoconvulsants along with a higher susceptibility to spontaneous seizures compared to age-matched wild-type mice [66]. Here, we show that this phenotype, although expected as part of APP/PS1 progression, is significantly impacted by innate immune cell regulation. We identified a novel regulator of microglia inflammatory function that, when deleted results, in early onset hyperexcitability and epileptogenesis in two mouse models of AD. We found that inducible loss of miR-155 in microglia results in early onset epileptogenesis, increased seizure severity, and reduced survival in the APP/PS1 AD model. Previous reports have also identified aberrant cortical excitability and altered theta oscillations in the 5xFAD mouse model [67]. More recently, Jubal et al., used two-photon calcium imaging to show an altered temporal distributions (burstiness) in the spontaneous activity of layer II/III visual cortex neurons 5xFAD mice, before plaque formation [68]. We specifically asked if miR-155 deletion in microglia resulted in early onset cortical hyperexcitability in 5xFAD mice. Increased interictal spikes were observed and significantly increased as early as 1 week after microglia miR-155 deletion, suggesting that microglia mediated signaling involving miR-155 contributes to the early changes in cortical excitability that occur in this model. Additional mechanisms can contribute to aberrant microglial synapse pruning that contribute to neuronal excitability. Altered purinergic signaling through P2Y12 and CD39, where Microglial CD39 and P2Y12 deletion leads to increased neuronal hyperexcitability and seizures [69, 70]. The link between the activity and or expression of CD39 and P2RY1, and how they are modified upon miR-155 deletion in amyloidosis mice, should be investigated further. Deletion of IL-18 is associated with lethal Picrotoxin-induced seizures in cerebral amyloidosis mice [71]. Future studies could focus on elucidating the link between miR-55 inflammatory regulation of microglia and alterations in IL-18. Our data fit with previous findings and support the hypothesis that the cellular functions of microglia extend to the restructuring of the already vulnerable circuitry in the context of amyloidosis in mouse models of AD, and that these functions converge on pathways modulated by miR-155. We identified that early onset hyperexcitability and spontaneous death after induced loss of miR-155 in microglia in APP/PS1 mice resulted from early onset epileptogenesis. Interestingly, constitutive deletion of miR-155 in both microglia and macrophages did not show alterations in overall survival of the APP/PS1 line, suggesting compensatory mechanisms at play to circumvent the loss of miR-155. Therefore, microglia functions in response to amyloid are regulated by miR-155 (e.g., phagocytosis) [28] and contribute to circuitry vulnerability and seizures in AD pathophysiology.

To our knowledge, our work is the first description of the conditional loss of miR-155 specifically in microglia in vivo resulting in early onset aberrant neuronal excitability, epileptogenesis, and mortality in a mouse model of AD. Studies focused on microglia-mediated synaptic loss have mainly focused on the engulfment of pre- and post-synaptic markers. In AD and age-related macular degeneration (AMD) mouse models, microRNAs miRNA-146a and miRNA-155 are progressively upregulated and hypothesized to drive inflammatory neurodegeneration through synaptic stripping [72]. In microglia isolated from the 5xFAD brain after miR-155 deletion, we found a significant increase in the levels of excitatory, VGLUT1-marked synapses internalized at the onset of aberrant cortical excitability (Fig. 7D, E). We also observed lower internalized levels of synaptic VGAT (Fig. 7B, C). This is crucial, as a hyperexcitable circuitry may seem paradoxical if not fully elucidated. This was observed in the pathophysiology of Dravet Syndrome where a loss of excitatory to inhibitory synaptic neurotransmission leads to epilepsy [38, 73]. We monitored the 5xFAD cohort for a short timeframe post miR-155 deletion in microglia, and our analysis of synaptic internalization employed a limited selection of excitatory and inhibitory markers. Future studies should focus on the circuitry-level changes of excitatory and inhibitory markers to further dissect the role of microglia-mediated synaptic stripping in the context of miR-155 deficient microglia and its impact on the hyperexcitable 5xFAD and APP/PS1 brain.

## Conclusion

Our study underlines the significance of miR-155 in microglia functions in AD pathophysiology and reveals its pleiotropic impact on circuitry homeostasis. Our findings provide the first evidence that miR-155 is a critical modulator and regulator of microglia functions and microglia-mediated synaptic homeostasis in early onset cortical hyperexcitability, spontaneous seizures, and death in amyloidosis models of AD.

## Supplementary Information

**Additional file 1: Figure S1. **Ratio of microglia to total cells isolated with ex vivo FACS. We found no significant difference in microglia counts (per total live cells) between groups. (Stats: 2-way ANOVA with Tukey’s correction for multiple comparisons, main effect of genotype, *p* = 0.3923).

**Additional file 2: Figure S2. **Changes in DAM profile and mature microglia markers in the APP/PS1 brain at 6 months of age with microglia-specific miR-155 deletion. We did not observe significant differences in Iba-1 (A: Ordinary one-way ANOVA with Sidak’s correction for multiple comparisons, p = 0.4366), Cst3 (C: Ordinary one-way ANOVA with Sidak’s correction for multiple comparisons, p = 0.7507) or Hexb (D: Ordinary one-way ANOVA with Sidak’s correction for multiple comparisons, *p* = 0.6188) between our three different conditions. We did, however, observe a significant decrease in Tmem119 in APP/ PS1 microglia relative to control (B: Ordinary one-way ANOVA with Sidak’s correction for multiple comparisons, p = 0.0266, Control v. APP/PS1: *p* = 0.0251). This decrease was maintained in the APP/PS1 MG miR-155 CKO group, but less pronounced and was not statistically significant (*p* = 0.1921).

**Additional file 3: Figure S3. **We did not observe a significant difference in the levels of soluble Aβ_1-42_ and Aβ_1-40_ in cortical lysates. Quantification of soluble Aβ_1-42_ and Aβ_1-40_ using Luminex from lysates of cortex with Luminex. There were no differences in soluble Aβ_1-42_ (two-tailed unpaired t-test, *p* = 0.3568) and Aβ_1-40_ (two-tailed unpaired t-test, *p* = 0.7505) levels detected with microglia-specific miR-155 deletion.

**Additional file 4: Figure S4. **Levels of pTau are not high in the APP/PS1 mouse model. We quantified the levels of pTau using Luminex bead-based assays. We did not observe a significant increase or decrease of pTau upon microglia-specific miR-155 deletion in A) the cortex (two tailed unpaired t-test, *p* = 0.7858) or B) hippocampus (two-tailed unpaired t-test, *p* = 0.1927) of APP/PS1 mice at 6 months of age.

**Additional file 5: Figure S5.** In situ analysis of Aβ fragments after conditional miR-155 deletion from microglia in 5xFAD mice suggests microglia. Two week post-conditional miR-155 deletion mice were sacrificed, and brains were post-fixed, then stained for Aβ (6E10) microglia (Iba-1). Detection of fibrillar Aβ fragments is sparse, and no plaque pathology is observed at 10 weeks of age.

**Additional file 6: Table S1.** Pairwise comparisons using Log-Rank test results for survival analysis.

## Data Availability

All data generated or analyzed during this study are included in this published article [and its additional information files].

## Abbreviations

AD: Alzheimer’s disease
MG: Microglia
APP: Amyloid precursor protein
PSEN1: Presenilin 1
PSEN2: Presenilin 2
EOFAD: Early onset familial Alzheimer’s disease
LOAD: Late onset sporadic Alzheimer’s disease
Aβ: Amyloid beta
TLR: Toll-like receptors
TREM2: Triggering receptors expressed on myeloid cells 2
CX3CR1: Receptor for the C–X3–C chemokine fractalkine
miRNA: MicroRNA
ECoG: Electrocorticography
EMG: Electromyography
PFA: Paraformaldehyde
PBS: Phosphate buffered saline
TBST: Tris buffered saline
VGAT: Vesicular GABA transporter
VGLUT: Vesicular Glutamate transporter
TLE: Temporal lobe epilepsy
CKO: Conditional knock-out
