# Supplementary material for: Microglia specific deletion of miR-155 in Alzheimer’s disease mouse models reduces amyloid-β pathology but causes hyperexcitability and seizures
Source: J Neuroinflammation. 2023 Mar 7;20:60. doi: 10.1186/s12974-023-02745-6 (PMC9990295; doi:10.1186/s12974-023-02745-6)
Supplement: Supplementary file 1 — Additional file 1: Figure S1. Ratio of microglia to total cells isolated with ex vivo FACS. We found no significant difference in microglia counts (per total live cells) between groups. (Stats: 2-way ANOVA with Tukey’s correction for multiple comparisons, main effect of genotype, p = 0.3923). [file 12974_2023_2745_MOESM1_ESM.pdf]

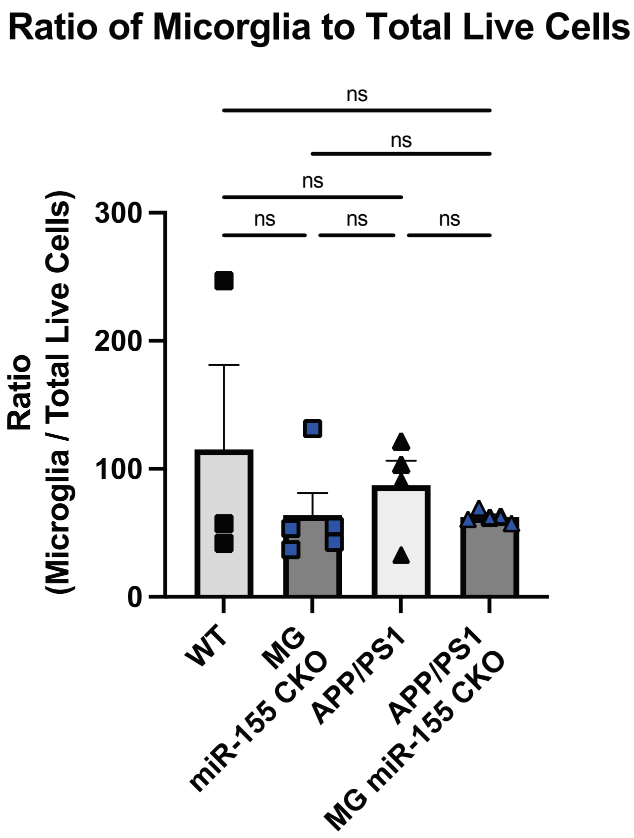

Supplemental Figure 1: Ratio of microglia to total cells isolated with ex vivo FACS. We found no significant difference in microglia counts (per total live cells) between groups (2-way ANOVA with Tukey's correction for multiple comparisons, main effect of genotype, $p = 0.3923$).
