# Supplementary material for: Microglia specific deletion of miR-155 in Alzheimer’s disease mouse models reduces amyloid-β pathology but causes hyperexcitability and seizures
Source: J Neuroinflammation. 2023 Mar 7;20:60. doi: 10.1186/s12974-023-02745-6 (PMC9990295; doi:10.1186/s12974-023-02745-6)
Supplement: Supplementary file 2 — Additional file 2: Figure S2. Changes in DAM profile and mature microglia markers in the APP/PS1 brain at 6 months of age with microglia-specific miR-155 deletion. We did not observe significant differences in Iba-1 (A: Ordinary one-way ANOVA with Sidak’s correction for multiple comparisons, p = 0.4366), Cst3 (C: Ordinary one-way ANOVA with Sidak’s correction for multiple comparisons, p = 0.7507) or Hexb (D: Ordinary one-way ANOVA with Sidak’s correction for multiple comparisons, p = 0.6188) between our three different conditions. We did, however, observe a significant decrease in Tmem119 in APP/ PS1 microglia relative to control (B: Ordinary one-way ANOVA with Sidak’s correction for multiple comparisons, p = 0.0266, Control v. APP/PS1: p = 0.0251). This decrease was maintained in the APP/PS1 MG miR-155 CKO group, but less pronounced and was not statistically significant (p = 0.1921). [file 12974_2023_2745_MOESM2_ESM.pdf]

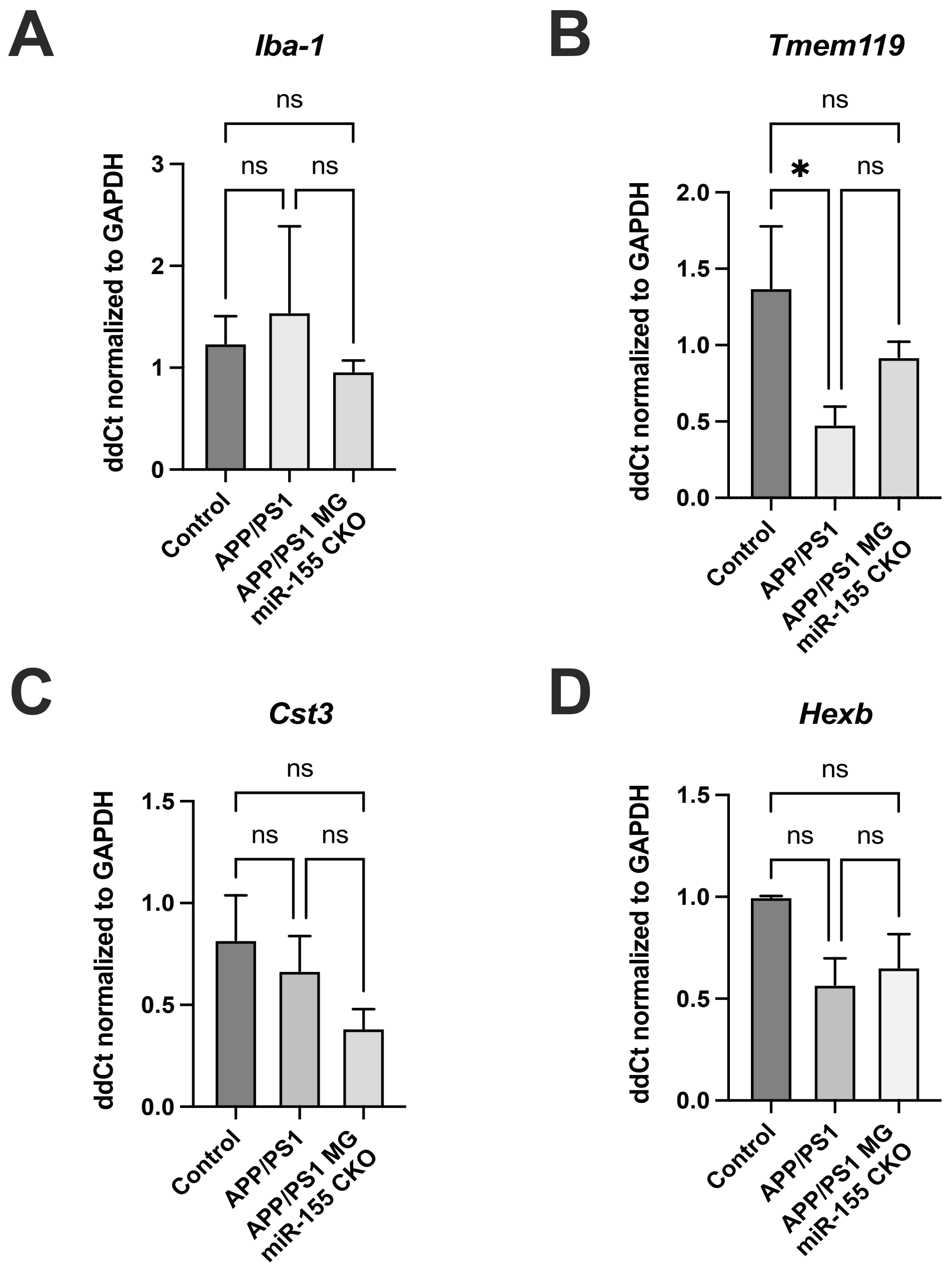

Supplemental Figure 2: Changes in DAM profile and mature microglia markers in the APP/PS1 brain at 6 months of age with microglia-specific miR-155 deletion. We did not observe significant differences in *Iba-1* (**A:** Ordinary one-way ANOVA with Sidak's correction for multiple comparisons, $p = 0.4366$), *Cst3* (**C:** Ordinary one-way ANOVA with Sidak's correction for multiple comparisons, $p = 0.7507$) or *Hexb* (**D:** Ordinary one-way ANOVA with Sidak's correction for multiple comparisons, $p = 0.6188$) between our three different conditions. We did, however, observe a significant decrease in *Tmem119* in APP/PS1 microglia relative to control (**B:** Ordinary one-way ANOVA with Sidak's correction for multiple comparisons, $p = 0.0266$, Control v. APP/PS1: $p = 0.0251$). This decrease was maintained in the APP/PS1 MG miR-155 CKO group, but less pronounced and was not statistically significant ($p = 0.1921$).
