# Supplementary material for: Microglia specific deletion of miR-155 in Alzheimer’s disease mouse models reduces amyloid-β pathology but causes hyperexcitability and seizures
Source: J Neuroinflammation. 2023 Mar 7;20:60. doi: 10.1186/s12974-023-02745-6 (PMC9990295; doi:10.1186/s12974-023-02745-6)
Supplement: Supplementary file 3 — Additional file 3: Figure S3. We did not observe a significant difference in the levels of soluble Aβ1-42 and Aβ1-40 in cortical lysates. Quantification of soluble Aβ1-42 and Aβ1-40 using Luminex from lysates of cortex with Luminex. There were no differences in soluble Aβ1-42 (two-tailed unpaired t-test, p = 0.3568) and Aβ1-40 (two-tailed unpaired t-test, p = 0.7505) levels detected with microglia-specific miR-155 deletion. [file 12974_2023_2745_MOESM3_ESM.pdf]

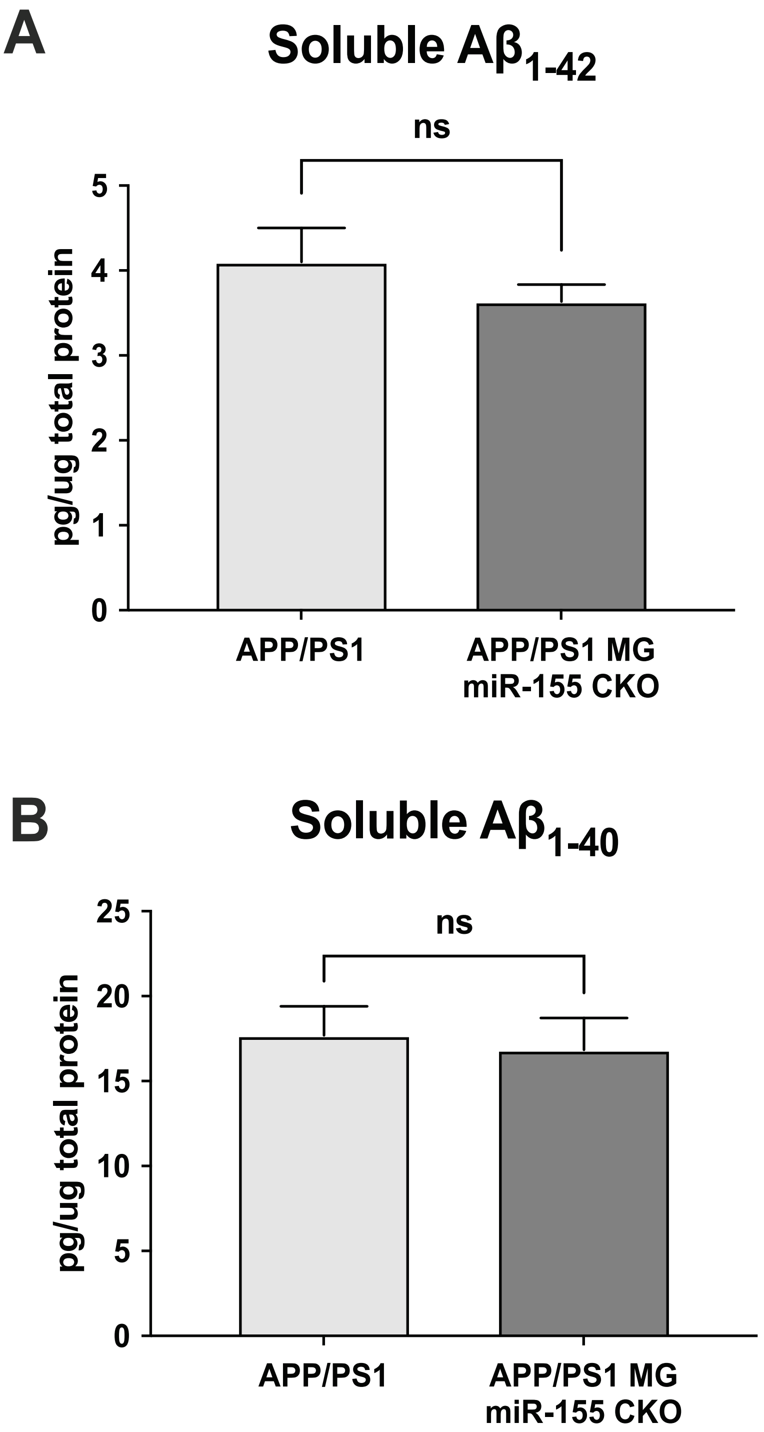

Supplemental Figure 3: We did not observe a significant difference in the levels of soluble $A\beta_{1\text{-}42}$ and $A\beta_{1\text{-}40}$ in cortical lysates. Quantification of soluble $A\beta_{1\text{-}42}$ and $A\beta_{1\text{-}40}$ using Luminex from lysates of cortex with Luminex. There were no differences in soluble $A\beta_{1\text{-}42}$ (two-tailed unpaired t-test, $p = 0.3568$) and $A\beta_{1\text{-}40}$ (two-tailed unpaired t-test, $p = 0.7505$) levels detected with microglia-specific miR-155 deletion.
