# Supplementary material for: Microglia specific deletion of miR-155 in Alzheimer’s disease mouse models reduces amyloid-β pathology but causes hyperexcitability and seizures
Source: J Neuroinflammation. 2023 Mar 7;20:60. doi: 10.1186/s12974-023-02745-6 (PMC9990295; doi:10.1186/s12974-023-02745-6)
Supplement: Supplementary file 4 — Additional file 4: Figure S4. Levels of pTau are not high in the APP/PS1 mouse model. We quantified the levels of pTau using Luminex bead-based assays. We did not observe a significant increase or decrease of pTau upon microglia-specific miR-155 deletion in A) the cortex (two tailed unpaired t-test, p = 0.7858) or B) hippocampus (two-tailed unpaired t-test, p = 0.1927) of APP/PS1 mice at 6 months of age. [file 12974_2023_2745_MOESM4_ESM.pdf]

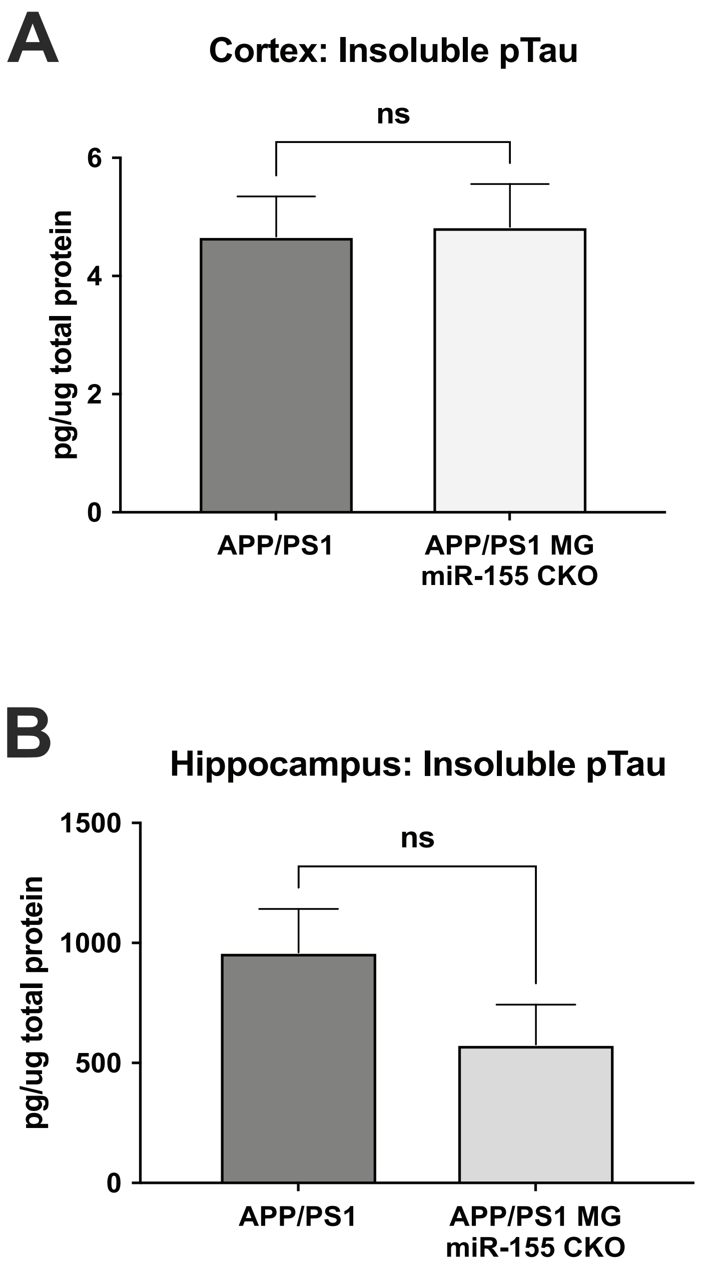

Supplemental Figure 4: **Levels of pTau are not high in the APP/PS1 mouse model.** We quantified the levels of pTau using Luminex bead-based assays. We did not observe a significant increase or decrease of pTau upon microglia-specific miR-155 deletion in **A)** the cortex (two-tailed unpaired t-test, $p = 0.7858$) or **B)** hippocampus (two-tailed unpaired t-test, $p = 0.1927$) of APP/PS1 mice at 6 months of age.
