# Supplementary material for: Microglia specific deletion of miR-155 in Alzheimer’s disease mouse models reduces amyloid-β pathology but causes hyperexcitability and seizures
Source: J Neuroinflammation. 2023 Mar 7;20:60. doi: 10.1186/s12974-023-02745-6 (PMC9990295; doi:10.1186/s12974-023-02745-6)
Supplement: Supplementary file 5 — Additional file 5: Figure S5. In situ analysis of Aβ fragments after conditional miR-155 deletion from microglia in 5xFAD mice suggests microglia. Two week post-conditional miR-155 deletion mice were sacrificed, and brains were post-fixed, then stained for Aβ (6E10) microglia (Iba-1). Detection of fibrillar Aβ fragments is sparse, and no plaque pathology is observed at 10 weeks of age. [file 12974_2023_2745_MOESM5_ESM.pdf]

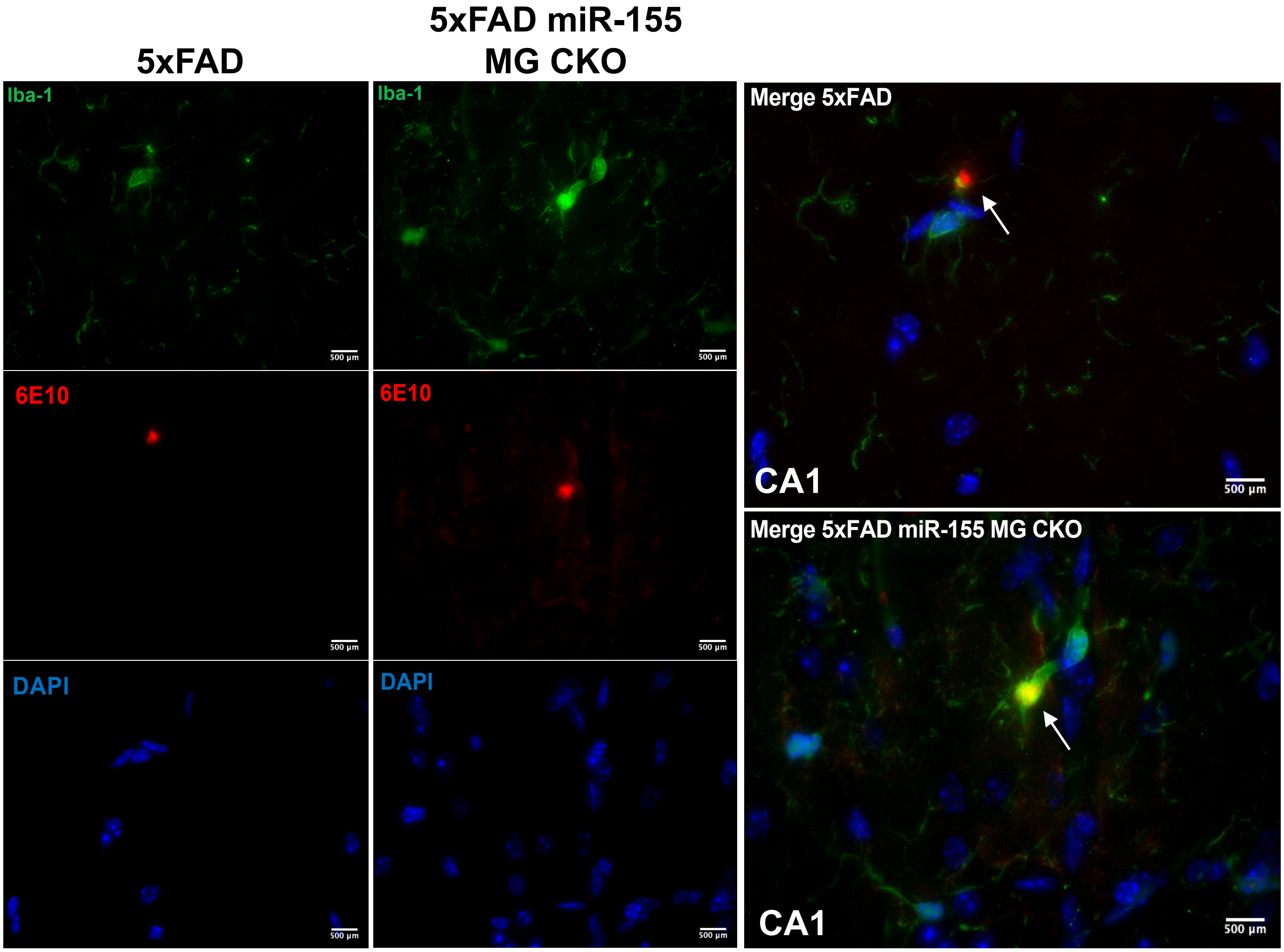

Supplemental Figure 5: In situ analysis of Aβ fragments after conditional miR-155 deletion from microglia in 5xFAD mice suggests microglia. Two weeks post conditional miR-155 deletion mice were sacrificed, and brains were post-fixed, then stained for Aβ (6E10) microglia (Iba-1). Detection of fibrillar Aβ fragments is sparse, and no plaque pathology is observed at 10 weeks of age.
