# Supplementary material for: Microglia specific deletion of miR-155 in Alzheimer’s disease mouse models reduces amyloid-β pathology but causes hyperexcitability and seizures
Source: J Neuroinflammation. 2023 Mar 7;20:60. doi: 10.1186/s12974-023-02745-6 (PMC9990295; doi:10.1186/s12974-023-02745-6)
Supplement: Supplementary file 6 — Additional file 6: Table S1. Pairwise comparisons using Log-Rank test results for survival analysis. [file 12974_2023_2745_MOESM6_ESM.pdf]

Supplemental Table-1: Pairwise comparisons using Log-Rank test results for survival analysis

	APP/PS1	APP/PS1 MG miR-155 CKO	APP/PS1 MG/MO miR-155 KO	Control	MG miR-155 CKO
APP/PS1 MG miR-155 CKO	***0.0463***	−	−	−	−
APP/PS1 MG/MO miR-155 KO	0.5965	0.0065	−	−	−
Control	1.5000E-05	9.0000E-11	0.0005	−	−
MG miR-155 CKO	2.8000E-05	2.3000E-10	0.0007	1.0000	−
MG/MO miR-155 KO	0.0127	0.0001	0.0325	1.0000	1.0000
